# Presence of Thioxanthones and Their Metabolites in Human Urine and Human Exposure Assessment

**DOI:** 10.3390/toxics13070535

**Published:** 2025-06-26

**Authors:** Lin Gao, Ling Zhang, Lisha Zhou, Fangfang Ren, Hangbiao Jin, Xiaoyu Wu

**Affiliations:** 1 Taizhou Central Hospital (Taizhou University Hospital), Taizhou University, Taizhou 318000, China; 2 School of Pharmaceutical Sciences, Taizhou University, Taizhou 318000, China; 3 Key Laboratory of Microbial Technology for Industrial Pollution Control of Zhejiang Province, College of Environment, Zhejiang University of Technology, Hangzhou 310032, China

**Keywords:** thioxanthones, 2-ITX, DETX, human urine, exposure assessment

## Abstract

Given the widespread environmental presence of thioxanthones (TXs), a class of commonly used photoinitiators, great concerns have been raised regarding their potential human exposure and associated health risks. However, a comprehensive understanding of the total burden of human exposure to these compounds remains limited. To address this gap, the current study collected urine samples from 211 healthy adults in Taizhou City, China, and, for the first time, analyzed the presence of TXs and their metabolites in human urine. The findings revealed that 2-ITX (2-isopropylthioxanthone) and DETX (2,4-diethylthioxanthone) were more frequently detected than other TXs, displaying the detection frequencies of 82% and 79%, respectively, in human urine. Measured mean levels of 2-ITX and DETX were 0.66 ng/mL and 0.51 ng/mL, respectively, in human urine. Female participants (0.67 ± 0.24 ng/mL) demonstrated higher (*p* < 0.01) urinary concentrations of DETX than male participants (0.42 ± 0.19 ng/mL). Human urinary levels of 2-isopropy1-10-oxothioxanthen-9-one (*p* = 0.011), 2-diisopropy1-10,10-dioxothioxanthen-9-one (*p* < 0.01), and DETX (*p* = 0.011) were negatively correlated with the age of individual participants. The calculated mean daily exposure value of 2-ITX (240 ng/kg bw/day) was much (*p* < 0.01) higher than that of DETX (151 ng/kg bw/day) for the participants. This study offers foundational information on human exposure to TXs, contributing to future environmental health research and the development of human exposure management strategies.

## 1. Introduction

Photoinitiators (PIs), a group of emerging aromatic organic pollutants, are widely utilized in various kinds of photopolymerization systems [1,2,3]. During photopolymerization, PIs generate reactive species through bond cleavage and hydrogen abstraction mechanisms, producing free radicals that serve as initiators for the polymerization reaction [4,5]. These PIs are predominantly incorporated into photosensitive materials such as Ultraviolet (UV)-curable resins, coatings, adhesives, and printing inks [6,7], which find extensive application across various industries for products ranging from surface primers and protective topcoats to pigmented paints and overprint varnishes [8]. As a global leader in the production of PIs, China manufactured approximately 29,000 metric tons of these compounds in 2007 [9]. Environmental studies have revealed significant PI emissions in China, with an estimated 307 tons released through UV-curable resin applications and 9.57 tons from food packaging materials every year [10,11]. Industry analysts anticipate substantial growth in PI manufacturing to meet the expanding market demand for photopolymerization-based products [4,12].

Thioxanthone derivatives (TXs) are a prominent subclass of photoinitiators, characterized by their thioxanthone core structure and exceptional photochemical properties. TXs represent one of the most extensively employed classes of PIs in industrial settings, owing to their remarkable photochemical characteristics and adaptability across diverse polymerization processes [1,4,13]. These TXs mainly included TX (thioxanthone), 2-ITX (2-isopropylthioxanthone), and DETX (2,4-diethylthioxanthone) [14,15,16]. Owing to their widespread use in commercial applications, studies have detected various TXs in food products, dust, nail cosmetics, and human blood [17,18,19,20]. This indicates a significant potential for human exposure to these chemicals through varying sources, which have attracted concerns about health risks, as evidenced by their cytotoxicity and endocrine-disrupting effects in vitro. After ingestion, 2-ITX and DETX in the human body can be metabolized into their respective sulfoxide and sulfone oxidation products [18]. Scientific evidence has suggested that exposure to TXs may potentially induce adverse health effects in humans. For example, in vitro studies using H295R cell models demonstrated that 2-ITX induced structural modifications in the phospholipid organization and significantly altered membrane rigidity characteristics [21]. 2-chloroisopropylthioxanthone (2-Cl-ITX) showed inhibitory effects on key human cytochrome P450 enzymes, specifically cytochrome P450 (CYP) 1A2 and CYP3A4 [22]. Toxicity assessments in rat hepatocytes indicated that DETX exhibited greater cytotoxicity than its structural analogs, including TX, 2-ITX, and 2-Cl-ITX [12,23]. Therefore, a comprehensive assessment of human exposure levels to TXs is crucial for establishing accurate risk evaluation frameworks.

The human body eliminates varying toxins through urine excretion, which serves as a primary detoxification mechanism [24,25,26]. The collection of urine specimens offers significant benefits as a straightforward and convenient method, rendering it an efficient option for large-scale research studies [27,28,29]. An examination of urine samples can be used to understand the changes in individuals’ exposure to environmental pollutants, offering valuable information about exposure patterns and associated health hazards [27,29,30]. As a result, urine analysis is considered a trustworthy approach for monitoring the intake of diverse contaminants in humans [31,32,33]. TXs are widely utilized in consumer goods, and studies have documented their presence in food packaging, indoor environments, and various environmental matrixes (e.g., indoor and outdoor dust, wastewater, river water, and sediment) [12]. The presence of these compounds in human blood from the general populations and pregnant women further highlights notable human TX exposure [18,34]. However, the extent of human TX exposure is not yet fully understood, necessitating additional research to clarify these aspects. To the best of our knowledge, this is the first study effort to identify and quantify TXs, as well as metabolites of 2-ITX and DETX, in human urine.

To address this research gap, urine samples were collected from healthy general adults residing in Taizhou City, China, and analyzed for the presence of five TXs, along with two metabolites of 2-ITX and two metabolites of DETX. The present study additionally investigated the potential gender- and age-based variations in urinary concentrations and the distribution patterns of the detected compounds. Additionally, the total daily intake of TXs was estimated using the measured urinary levels. The findings offer foundational data on exposure levels to TXs among the general population, which is crucial for evaluating the possible hazards related to human contact with these substances.

## 2. Materials and Methods

### 2.1. Standard Chemicals

The target analytes in this study included five TXs, two metabolites of 2-ITX, and two metabolites of DETX. Native standards for TX, DETX, 2-Cl-TX, 2-ITX, and 2-TF-TX (2-trifluoromethylthioxanthone) were supplied by Sigma-Aldrich (New York, NY, USA) and Aladdin (Shanghai, China). The native standards for metabolites of 2-ITX and DETX (solid phase with purities exceeding 97%), including 2-ITX-O (2-isopropy1-10-oxothioxanthen-9-one), 2-ITX-O_2_ (2-diisopropy1-10,10-dioxothioxanthen-9-one), DETX-O (2,4-diethyl-10-oxothioxanthen-9-one), and DETX-O_2_ (2,4-diethy1-10,10-dioxothioxanthen-9-one), were custom synthesized by Yuhao Chemical (Hangzhou, China) based on established methods [18,35]. The isotopically labeled standard (2-ITX-D_7_) was purchased from Sigma-Aldrich (New York, NY, USA). Additional details regarding all standards are provided in the Supporting Information (Supplementary Materials, Table S1 and Figure S1).

### 2.2. Study Participants and Urine Sample Collection

This study enrolled 211 healthy adult participants from the population of Taizhou City during July and September 2024. Geographically positioned within eastern China’s regional basin, Taizhou has emerged as a metropolitan hub with a demographic profile surpassing ten million permanent residents. Fasting morning urine specimens were collected from all participants under the supervision of qualified nursing staff, and these samples were preserved in PP tubes (volume 15 mL; Accin Medical, Shanghai, China). All of the obtained samples were stored at a temperature of −60 °C until analysis. The present study included 123 male and 88 female participants. Their average ages were 47 ± 14 years and 42 ± 14 years, respectively, and all had lived in Taizhou City for over two years. The average BMI for male and female participants was 26 ± 6.9 kg/m^2^ and 23 ± 5.0 kg/m^2^, respectively. Most of the participants had their annual household income fall below 150,000 CNY. All enrolled individuals denied using TX-containing medications during recruitment. Detailed demographic data of the recruited subjects are provided in Supplementary Materials, Table S2. The ethics committee of Taizhou Central Hospital approved the research protocol of this study.

### 2.3. Extraction of Human Urine Samples

The extraction of target analytes from human urine samples was conducted following established methods [18], with certain adjustments. Briefly, a volume of 2.0 mL of human urine specimens was fortified with 2-ITX-D_7_ (at 5.0 ng) for analytical quantification. These samples were then combined with 2.0 mL of methyl tert-butyl ether (MTBE). The mixtures were vortexed for 1 min, shaken at 245 rpm for 45 min, and centrifuged at 4500× *g* for 8 min. The supernatant was gathered, and the extraction procedure was performed two additional times. All supernatants were pooled (approximately 6 mL), evaporated under a nitrogen stream, and reconstituted in 2 mL of methanol. These sample extracts were further purified using the Carb-GCB cartridges (3 mL/500 mg; KEPNO; Shandong, China), which were preconditioned with methanol (3 mL). These purified solvents were evaporated to dryness and reconstituted in 50 μL of methanol for the subsequent instrumental analysis.

### 2.4. Instrumental Analysis

The analytes in the sample extracts were identified and quantified utilizing an ultrahigh-performance liquid chromatography system (UPLC) paired with a Xevo triple–quadrupole tandem mass spectrometer (MS/MS; Waters, Milford, MA, USA). For the separation process, an ACQUITY BEH C18 column (2.1 × 100 mm, 1.7 μm) was employed in the UPLC system. This column was maintained at 35 °C, with the mobile phase consistently delivered at 0.3 mL/min. The gradient elution started with a mixture of 10% methanol and 90% water (both containing 0.1% formic acid), which was held for 1 min. This was followed by a linear increase to 95% methanol over 10 min, and the final composition was maintained for another 1 min before returning to the initial condition. The analysis was conducted using electrospray ionization in positive ion mode. The ionization source temperature was maintained at 125 °C, while the desolvation temperature was set to 390 °C. The desolvation gas flow rate was fixed at 600 L/h, and the cone gas flow rate was adjusted to 100 L/h. Specific parameters for multiple reaction monitoring (MRM) can be found in Supplementary Materials, Table S3.

### 2.5. Human Daily Excretion Estimation

In the current study, the daily exposure (DE, ng/kg body weight/day) to 2-ITX and DETX was estimated for the general population based on the measured concentrations of these compounds and their metabolites in human urine. The calculation of DE was performed using an equation derived from methodologies established in prior studies [36,37].

$$DE=\sum \frac{C_{urine}{\times V}_{urine}\times M_{parent}}{W\times M_{metabo}}$$

where the variable *C_urine_* corresponds to the measured concentrations (ng/mL) of 2-ITX and DETX in human urine samples; *V_urine_* represents the daily urine excretion volume, which was set at 1800 mL/day for Chinese adults based on values reported in prior research [37]; *W* denotes the body weight (kg) of the participants, as recorded during the questionnaire survey; *M_parent_* refers to the molecular weight of the parent compounds (2-ITX and DETX); and *M_metabo_* indicates the molecular weight of their respective metabolites (*2-ITX-O* and *2-*ITX-O_2_ for 2-ITX; DETX-O and DETX-O_2_ for DETX). The estimation of DE is limited by the use of morning spot urine samples, as urinary levels of target analytes may vary throughout the day. However, previous research had considered early morning urine to be a practical matrix for assessing human exposure [38,39].

### 2.6. QA/QC

Several quality assurance measures were implemented throughout the analyzing processes for the target analytes. Disposable glassware, meticulously cleaned with pure methanol, was employed during urine sample extraction. Procedural blank samples were systematically analyzed alongside every batch of 10 human urine samples. To address instrumental carryover, pure methanol injections were performed and analyzed after every 10 urine samples. No target analytes were detected in field blank or procedural blank samples, confirming the absence of contamination. To monitor the consistency of the instrument’s performance, a calibration standard with a concentration of 15 ng/mL for each target analyte was systematically analyzed following every set of 10 samples.

To measure the levels of target analytes in human urine, this study utilized an internal standard methodology [40]. Calibration curves (linearity range 0.10–100 ng/mL) were constructed in methanol for each target analyte, with each curve comprising at least six concentration points and demonstrating high linearity (R^2^ > 0.995). The limits of detection (LODs) for the target analytes were defined as the lowest urinary concentrations capable of producing a signal-to-noise ratio of three. The limits of quantification (LOQs) for target analytes were defined as the lowest urinary concentrations capable of producing a signal-to-noise ratio of ten. The calculated LOQs ranged from 0.075 ng/mL (2-ITX-O) to 0.34 ng/mL (DETX-O) for target analytes. The calculated LODs ranged from 0.025 ng/mL (2-ITX-O) to 0.11 ng/mL (DETX-O) for target analytes (see Supplementary Materials, Table S4). To determine the extraction efficiency, human urine samples were prepared with added analytes at three specific concentrations (0.50 ng/mL, 5.0 ng/mL, and 50 ng/mL) and analyzed using the developed method. The extraction recoveries for the target analytes ranged from 83% (TX) to 113% (2-TF-TX) (see Supplementary Materials, Table S4). Notably, no adjustments were made to the reported urinary concentrations of target analytes based on the extraction recovery rates. To evaluate matrix effects, we compared the signal responses of standards spiked into blank human urine with those prepared in pure methanol [41]. The results showed matrix effects between 95% and 108% for all target analytes. Additionally, the reliability of the measurement technique was assessed by conducting intra- and inter-day variability studies, employing human urine samples spiked with target analytes at two concentration levels (0.50 ng/mL and 25 ng/mL). The intra-day variability ranged from 4.0% to 17%, while the inter-day variability ranged from 8.9% to 15%.

### 2.7. Statistical Analysis

In this study, statistical analysis was exclusively performed on target analytes that demonstrated detection frequencies exceeding 50%. The normality of analyte concentration distributions in human urine specimens was examined through the Kolmogorov–Smirnov normality test. Interrelationships between various target analyte concentrations in the obtained urine samples were investigated based on the calculated Spearman’s rank correlation coefficient. To evaluate concentration variations, the Mann–Whitney *U* test was implemented for the specific comparisons: (1) concentration differences among distinct target analytes in urine samples, (2) gender-based variations in urinary analyte concentrations between male and female participants, and (3) age-related differences in urinary concentrations of various target analytes. All statistical computations were conducted using SPSS Statistics software (Version 26.0; IBM, Armonk, New York, NY, USA), with statistical significance established at a two-tailed *p*-value threshold of less than 0.05.

## 3. Results and Discussion

### 3.1. TXs in Human Urine

**Figure 1:**
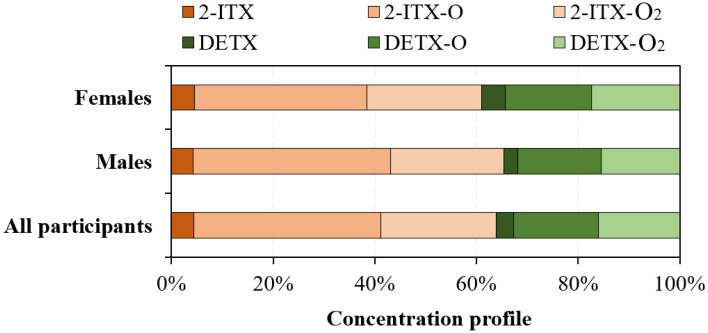
Concentration profiles of 2-ITX, DETX, and their metabolites in human urine samples from female participants, male participants, and all participants.

Analyzing TXs in collected human urine showed that 2-ITX and DETX were more frequently detected than 2-TF-TX, 2-Cl-TX, and TX, displaying detection frequencies of 82% and 79%, respectively (Table 1). The detection frequencies of 2-TF-TX, 2-Cl-TX, and TX were lower than 20% in human urine samples. The total concentration of all detected TXs (∑TXs) was in the range of <LOD–6.2 ng/mL (mean 1.2 ng/mL, median 1.1 ng/mL). 2-ITX (mean 0.66 ng/mL, range < LOD–3.1 ng/mL) and DETX (0.51 ng/mL, <LOD–5.9 ng/mL) were the predominant TXs in human urine, accounting for an average of 55% and 45% of ∑TXs, respectively (Figure 1).

To our knowledge, no previous studies have examined these TXs in human urine samples. Monitoring studies on indoor dust from residential houses, printing shops, and nail salons in China also showed that 2-ITX and DETX were always the predominant TXs [11,20]. TXs in human blood from China and the United States were also dominated by 2-ITX and DETX [18,42]. This may suggest that 2-ITX and DETX were the most dominantly utilized TXs in varying consumer products. The high detection frequencies of 2-ITX and DETX in collected human urine, compared with other TXs (e.g., 2-TF-TX, 2-Cl-TX, and TX), may further imply their high bioavailability, bioaccumulation potential, or higher exposure levels in the general population. Notably, previous studies focused on monitoring TXs in various environmental samples and human blood did not include 2-TF-TX [12]. To our knowledge, this is the first study revealing the presence of 2-TF-TX in human urine. This may suggest that humans were exposed to a broader range of TXs than previously recognized. Future monitoring studies should expand the scope to include 2-TF-TX and other potentially relevant TXs to better assess human exposure and associated health risks. In addition, concentrations of 2-ITX were not significantly (Spearman’s coefficient, ρ = 0.21, *p* = 0.18) correlated with that of DETX in collected human urine (Supplementary Materials, Table S5). This suggests that these two TXs may have different exposure sources, exposure pathways, or metabolic behaviors in the human body.

While our study provides the first biomonitoring data of TX in the urine of the general population, the potential health implications warrant careful consideration. In vitro studies using H295R adrenocortical cells have shown that 2-ITX (at concentrations as low as 1 μM) could significantly alter cortisol and estradiol production by interfering with steroidogenic enzymes [21]. This is particularly relevant given that the upper range of urinary 2-ITX levels in our study (3.1 ng/mL ≈ 12 nM) approaches biologically active concentrations when accounting for tissue accumulation. Comparative studies indicated that DETX exhibited greater cytotoxicity (IC50 = 8.2 μM) than other TX analogs in rat hepatocytes [23]. The observed urinary DETX levels may represent significant exposure when considering chronic low-dose effects and potential bioaccumulation.

### 3.2. Metabolites of 2-ITX and DETX in Human Urine

Metabolites 2-ITX, 2-ITX-O, and 2-ITX-O_2_ were detected in 76% and 77% of human urine samples, respectively, displaying urinary concentrations of <LOD–29 ng/mL (mean 5.5 ng/mL) and <LOD–27 ng/mL (mean 3.4 ng/mL), respectively (Table 1). Comparatively, the mean urinary levels of 2-ITX-O and 2-ITX-O_2_ were much (*p* < 0.01) higher than that of 2-ITX. This is different from that previously observed in human serum from the United States, which showed higher plasma levels of 2-ITX (geometric mean 35 pg/mL) than 2-ITX-O (6.8 pg/mL) and 2-ITX-O_2_ (13 pg/mL) [18]. In addition, we found that urinary concentrations of 2-ITX were significantly correlated with those of 2-ITX-O (ρ = 0.52, *p* = 0.002) and 2-ITX-O_2_ (ρ = 0.60, *p* < 0.01) (Supplementary Materials, Table S5). We speculate that 2-ITX was metabolized in the liver or other tissues into its oxidative metabolites, 2-ITX-O and 2-ITX-O_2_, which are more water soluble and thus more readily excreted in urine [18]. This would explain why the urinary levels of 2-ITX-O and 2-ITX-O_2_ were significantly higher than that of the parent compound (2-ITX). The observed metabolite profile aligns with known toxicokinetic pathways of TXs. In vitro studies demonstrated that 2-ITX and DETX undergo rapid CYP450-mediated oxidation to sulfoxide and sulfone metabolites in human liver microsomes, with minimal glucuronidation [18]. While human half-life data are unavailable, rat studies indicated plasma elimination half-lives of 4–6 h for DETX [23], suggesting its rapid clearance. In our study, the predominance of sulfoxide/sulfone metabolites may reflect the efficient Phase I metabolism and renal excretion, consistent with the physicochemical transition from lipophilic parents (log *K*_ow_ = 4.2 for 2-ITX) to hydrophilic metabolites (log *K*_ow_= 0.8 for 2-ITX-O_2_) [18]. This pattern implies that urinary metabolites likely represent recent exposure (within 24 h), though further pharmacokinetic studies are needed to confirm excretion kinetics of target analytes in humans.

The metabolites DETX-O and DETX-O_2_ were identified in 82% and 85% of human urine samples, respectively. Their mean urinary concentrations were 2.5 ng/mL (range < LOD–18 ng/mL) and 2.4 ng/mL (range < LOD–18 ng/mL), respectively. Comparatively, the mean urinary levels of DETX-O and DETX-O_2_ were much (*p* < 0.01) higher than that of DETX. Analyzing human serum from the United States reported higher plasma levels of DETX (geometric mean 63 pg/mL) than DETX-O (17 pg/mL) and DETX-O_2_ (9.6 pg/mL) [18]. This is plausible because DETX-O and DETX-O_2_ are more hydrophilic than DETX, which makes them more readily excreted in human urine. Correlation analysis showed that urinary concentrations of DETX were significantly correlated with those of DETX-O (ρ = 0.56, *p* = 0.018) but not with DETX-O_2_ (ρ = 0.20, *p* = 0.21). Urinary concentrations of DETX-O were also not significantly correlated with DETX-O_2_ (ρ = 0.15, *p* = 0.22). The metabolism of DETX (or DETX-O) into DETX-O_2_ had been previously demonstrated in the human liver S9 incubation model [18]. These data may suggest that, in addition to the metabolism of DETX (or DETX-O) into DETX-O_2_, humans may also be directly exposed to DETX-O_2_ through other sources, such as oral intake. Further studies are needed to validate this speculation.

### 3.3. Gender-Specific and Age-Specific Differences

**Figure 2:**
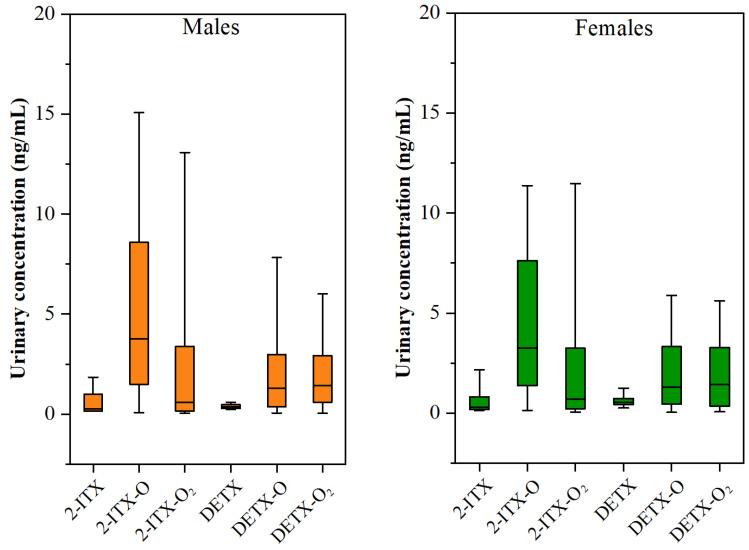
Concentrations of 2-ITX, DETX, and their metabolites in human urine samples from male participants and female participants.

**Figure 3:**
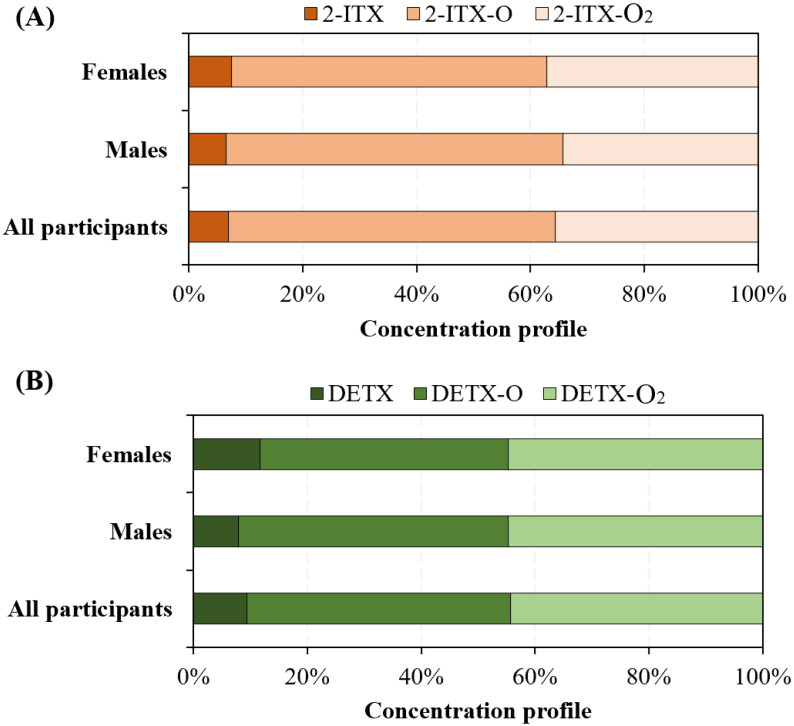
(**A**) Concentration profile of 2-ITX and its metabolites in human urine samples from female participants, male participants, and all participants. (**B**) Concentration profile of DETX and its metabolites in human urine samples from female participants, male participants, and all participants.

To examine the impact of gender on the urinary levels of target analytes, the collected human urine samples were categorized based on gender into male (*n* = 123) and female (*n* = 88) groups. The determined urinary concentrations of 2-ITX, DETX, and their metabolites for both male and female participants are shown in Figure 2. Comparison analysis showed a statistically significant difference (*p* < 0.05) in DETX levels, with female participants (0.67 ± 0.24 ng/mL) demonstrating higher urinary concentrations than the male participants (0.42 ± 0.19 ng/mL). Female participants may have higher exposure to DETX due to the use of certain consumer products containing this compound. For example, DETX has been demonstrated to be present in nail cosmetics that were more commonly used by women in China [20,43]. Other biological factors, such as body composition, hormone levels, or metabolic rates, also could influence the urinary excretion of DETX by the human body [44,45,46]. In addition, the urinary concentration profiles of 2-ITX and its metabolites are similar between male and female participants (Figure 1 and Figure 3A). Also, the urinary concentration profiles of DETX and its metabolites are similar between male and female participants (Figure 1 and Figure 3B).

**Figure 4:**
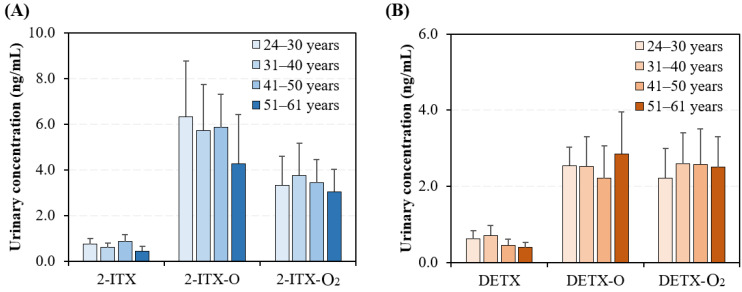
(**A**) Urinary concentrations (mean ± SD) of 2-ITX and its metabolites across the four human age groups. (**B**) Urinary concentrations (mean ± SD) of DETX and its metabolites across the four human age groups.

This research also explored potential variations in urinary concentrations and profiles of 2-ITX and its metabolites, as well as DETX and its metabolites, across participants of different age groups (Figure 4 and Figure S2). The results showed that the measured human urinary levels of 2-ITX-O (ρ = 0.54, *p* = 0.011), 2-ITX-O_2_ (ρ = 0.71, *p* < 0.01), and DETX (ρ = 0.56, *p* = 0.011) were negatively correlated with the age of individual participants. For example, the average urinary levels of DETX in participants showed a gradual decrease with age, dropping from 0.61 ± 0.22 ng/mL in the 24–30 years group (*n* = 49) to 0.39 ± 0.14 ng/mL in the 51–61 years group (*n* = 44). This observed trend can be attributed to multiple underlying factors, particularly age-related physiological changes and behavioral patterns [47,48]. A key consideration is the natural decline in renal function associated with aging [49,50], which may impair the metabolic clearance and urinary excretion of specific compounds, particularly 2-ITX-O and 2-ITX-O_2_, in older individuals. Lifestyle modifications commonly observed in older Chinese populations [51], including altered dietary preferences and decreased engagement with environments containing DETX-based products, may contribute to reduced exposure levels. Conversely, younger demographic groups demonstrate potentially elevated DETX exposure, potentially attributable to increased consumption of processed food products and more extensive utilization of personal care items, such as nail cosmetics, that may contain DETX derivatives [43]. These findings highlight the critical importance of incorporating age-specific parameters in exposure risk assessments and emphasize the need for further mechanistic studies to elucidate the biological and behavioral factors underlying these age-dependent exposure differentials.

### 3.4. Daily Exposure (DE) to 2-Cl-TX and DETX

Previously, Liu and Mabury [18] emphasized the necessity of considering their metabolites when assessing the burdens of human exposure to 2-ITX and DETX. So, in this study, we converted the measured human urinary levels of 2-ITX and DETX metabolites into equivalent concentrations of their parent compounds, enabling the calculation of the total exposure burden to these substances among study populations. The calculated mean DE of 2-ITX (240 ng/kg bw/day, range < 1.0–915 ng/kg bw/day) was much (*p* < 0.01) higher than that of DETX (151 ng/kg bw/day, range < 1.3–732 ng/kg bw/day) for the participants (Table 2). In addition, no significant differences in DE values were found between male and female participants for either 2-ITX (*p* = 0.22) or DETX (*p* = 0.47).

Several earlier studies had estimated the daily TX exposure amounts for different Chinese populations. For example, adult inhabitants in urban and rural regions of South China were found to ingest TXs through indoor dust at levels between 0.01 and 0.1 ng/kg bw/day based on 50th to 90th percentile values [11]. Female employees in Chinese nail salons were reported to consume two types of TXs via indoor dust in the amounts of 0.03–0.28 ng/kg bw/day, according to the 50th to 90th percentile estimates [20]. In the study by Shen et al., the estimated daily intake levels of 27 photoinitiators, which encompassed the target TXs, were determined to be 5.5 ng/kg bw/day at the median level and 45 ng/kg bw/day at the 95th percentile for general adults in China [43]. After comparison, we found that the human daily intake levels of TXs reported in these previous studies are substantially lower than the mean DE values of 2-ITX and DETX determined in this study. This discrepancy underscores the importance of investigating additional exposure pathways, such as dermal absorption, drinking water intake, and food consumption, to fully understand the population’s total TX exposure burden.

Currently, no established reference dose (RfD) or tolerable daily intake (TDI) values exist for 2-ITX or DETX due to limited toxicity studies. Benzophenone (BP), as a structurally similar photoinitiator with well-characterized toxicity (including endocrine disruption potential), serves as a conservative proxy for preliminary risk assessment. To assess possible risks, the calculated DE values of TXs in this study were compared with the RfD of BP, set at 30 μg/kg bw/day, following methodologies from prior research [11]. The DE values for 2-ITX and DETX were significantly below the RfD of BP, indicating that current 2-ITX and DETX exposure levels are unlikely to pose significantly immediate health risks to the general population. However, this finding should be approached with caution due to the lack of well-established toxicological thresholds for these TXs. Additionally, the potential for synergistic “cocktail” effects from simultaneous exposure to multiple TXs should be considered, as these interactions could potentially increase health risks. A thorough grasp of both individual and combined exposure risks is crucial to developing effective strategies to manage potential hazards.

### 3.5. Limitations of This Study

This study has several limitations that should be acknowledged. First, the participant cohort was drawn solely from an urban Chinese population, which may limit generalizability to other demographic or geographic groups. Second, while we focused on Phase I metabolites (e.g., 2-ITX-O and DETX-O_2_), potential Phase II conjugates (e.g., glucuronides) were not assessed due to the lack of analytical standards and enzymatic hydrolysis protocols validated for TXs. Third, although urinary metabolite levels provide integrated exposure estimates, they cannot identify dominant exposure pathways (e.g., food packaging vs. personal care products) without paired environmental measurements. Finally, while morning urine samples were widely used for biomonitoring, repeated sampling would better characterize intra-individual variability. Future studies should expand to multi-center cohorts, include conjugated metabolite analysis, and integrate environmental sampling to address these gaps.

## 4. Conclusions

This study provides the first comprehensive analysis of TXs and their metabolites in human urine. The high detection frequencies of 2-ITX and DETX, along with their oxidative metabolites, underscore the widespread human exposure to these photoinitiators, which are commonly used in various industrial and consumer products. Notably, this research is the first to identify and quantify 2-TF-TX in human urine, suggesting that human exposure to TXs may be more extensive than previously recognized. Future research should expand the scope of TX monitoring to include additional compounds and explore other exposure pathways, including dermal absorption, dietary intake, and inhalation. Gender-based variations were observed in urinary concentrations, with female participants showing higher levels of DETX. Age-related differences in excretion patterns were also evident for 2-ITX-O, 2-ITX-O_2_, and DETX. The daily exposure to 2-ITX and DETX was also estimated for participants. Moreover, mechanistic studies are needed to elucidate the metabolic pathways of TXs and their potential health impacts. Given the widespread use of TXs in consumer products, these findings highlight the need for ongoing monitoring and risk assessment to better understand the potential health impacts of these emerging pollutants.

## Figures and Tables

**Table 1:** Measured concentrations (ng/mL) of TXs and their metabolites in human urine (*n* = 211).

	Detection Frequency						
		Mean	Median	Min	25th	75th	Max
2-ITX	82%	0.66	0.72	<LOD	0.17	0.93	3.1
2-ITX-O	76%	5.5	3.8	<LOD	1.3	7.8	29
2-ITX-O_2_	77%	3.4	2.9	<LOD	0.19	12	27
DETX	79%	0.51	0.45	<LOD	0.29	3.9	5.9
DETX-O	82%	2.5	1.9	<LOD	0.37	8.4	18
DETX-O_2_	85%	2.4	2.4	<LOD	0.45	4.3	18
2-TF-TX	19%	NC *^a^*	<LOD	<LOD	<LOD	<LOD	8.6
2-Cl-TX	8.1%	NC *^a^*	<LOD	<LOD	<LOD	<LOD	2.9
TX	5.1%	NC *^a^*	<LOD	<LOD	<LOD	<LOD	0.60

*^a^* Note that “NC” means “not calculated”. Mean concentration was not calculated for analytes that were detected in less than 50% of the urine samples.

**Table 2:** Amount of human daily exposure to 2-ITX and DETX (ng/kg bw/day).

	Mean	Median	Min	25th	75th	Max
*All participants*						
2-ITX	240	176	<1.0	51	324	915
DETX	151	147	<1.3	19	295	732
*Female participants*						
2-ITX	248	195	<1.0	50	347	915
DETX	158	152	<1.3	20	302	731
*Male participants*						
2-ITX	219	192	<1.2	44	284	870
DETX	148	144	<1.3	14	328	708

## Data Availability

The data that support the findings of this study are available from the corresponding author upon reasonable request.

## Supplementary Materials

The following supporting information can be downloaded at https://www.mdpi.com/article/10.3390/toxics13070535/s1, Table S1: Abbreviations, CAS numbers, and full names of target analytes; Table S2: Demographic characteristics of participants (*n* = 211) recruited in this study; Table S3: MRM transitions of target compounds. MRM detection window for each compound was set at 50 m per second; Table S4: LODs and extraction recoveries of TXs in human urine; Table S5: Correlations in urinary concentrations between different analytes; Figure S1: Chemical structure of target TXs and their metabolites analyzed in this study; Figure S2: (A) Urinary concentration profiles of 2-ITX and its metabolites across the four human age groups. (B) Urinary concentration profiles of DETX and its metabolites across the four human age groups.
