# Supplementary material for: Presence of Thioxanthones and Their Metabolites in Human Urine and Human Exposure Assessment
Source: Toxics. 2025 Jun 26;13(7):535. doi: 10.3390/toxics13070535 (PMC12300941; doi:10.3390/toxics13070535)
Supplement: Supplementary file 1 [file toxics-13-00535-s001.zip › toxics-3659278-supplementary.pdf]

Table S1. Abbreviation, CAS Numbers, and Full Names of Target Analytes.

Full name	Abbreviation	CAS No.
**Thioxanthone**	TX	492-22-8
**2,4-Diethylthioxanthone**	DETX	82799-44-8
**2-Chlorothioxanthone**	2-Cl-TX	86-39-5
**2-Isopropylthioxanthone**	2-ITX	5495-84-1
**2-Trifluoromethylthioxanthone**	2-TF-TX	1693-28-3
**2-Isopropylthioxanthone-D_7**	2-ITX-D_7	N/A
**2-Isopropy1-10-oxothioxanthen-9-one**	2-ITX-O	N/A
**2-Diisopropy1-10,10-dioxothioxanthen-9-one**	2-ITX-O_2	N/A
**2,4-Diethyl-10-oxothioxanthen-9-one**	DETX-O	N/A
**2,4-Diethy1-10,10-dioxothioxanthen-9-one**	DETX-O_2	N/A

Table S2. Demographic Characteristics of Participants (*n* = 211) Recruited in This Study.

	***N* (%); Mean ± SD**
**Gender**	
Male participants	123 (58%)
Female participants	88 (42%)
**Age group**	
24−30 years	49 (23%)
31−40 years	50 (24%)
41−50 years	68 (32%)
51−61 years	44 (21%)
**BMI (kg/m^2)**	
Male participants	26 ± 6.9
Female participants	23 ± 5.0
**Education level**	
College	59 (28%)
High school	112 (53%)
Below high school	40 (19%)
**Annual household income (CNY)**	
< 80, 000	93 (44%)
80, 000−150, 000	97 (46%)
> 150, 000	21 (10%)

Table S3. MRM Transitions of Target Compounds. MRM Detection Window for Each Compound was Set at 50 m Second.

	Precursor ion (*m*/*z*)	Product ion (*m*/*z*)	Cone voltage (eV)	Collision energy (eV)	Retention time (min)
**TX**	213.2	184.1*	45	12	6.01
		152.0	48	15	
**2-Cl-TX**	247.2	212.1*	50	20	7.42
		184	50	22	
**2-ITX**	255.3	213.1*	55	15	8.29
		184	55	18	
**DETX**	269.3	241.1*	45	10	9.46
		213	45	14	
**2-TF-TX**	281.3	207.2*	50	11	8.11
		192.1	50	26	
**2-ITX-O**	271.1	239.1*	47	11	5.60
		254.2	47	11	
**2-ITX-O_2**	287.1	181.1*	40	19	4.66
		245.1	40	12	
**DETX-O**	285.2	253.1*	45	12	6.80
		268.1	45	12	
**DETX-O_2**	301.1	165.1*	50	25	5.07
		273.2	50	15	
**2-ITX-D_7**	262.2	214.1	55	30	8.28

* means the quantification ion.

Table S4. LODs and Extraction Recoveries of TXs in Human Urine.

	LODs (ng/mL)	Extraction recovery (%)					
		Spiked at 0.50 ng/mL (n = 5)		Spiked at 5.0 ng/mL (n = 5)		Spiked at 50 ng/mL (n = 5)	
		**Mean**	**SD**	**Mean**	**SD**	**Mean**	**SD**
**TX**	0.061	110	6	83	7	94	11
**2-Cl-TX**	0.065	101	10	106	4	99	6
**2-ITX**	0.048	102	8	93	4	93	9
**DETX**	0.065	99	6	91	11	94	7
**2-TF-TX**	0.104	98	11	95	6	113	5
**2-ITX-O**	0.025	91	10	106	7	106	6
**2-ITX-O_2**	0.026	112	5	83	10	102	9
**DETX-O**	0.107	97	6	89	9	89	8
**DETX-O_2**	0.076	95	9	99	10	89	8

Table S5. Correlations in Urinary Concentrations between Different Analytes.

	Spearman's rho	DETX	2-ITX-O	2-ITX-O_2	DETX-O	DETX-O_2
**2-ITX**	**Correlation Coefficient**	.210	.516**	.603**	-.092	-.033
	**Sig. (2-tailed)**	.180	.002	.000	.256	.680
**DETX**	**Correlation Coefficient**	1.000	-.096	.128	.559*	.204
	**Sig. (2-tailed)**	.	.254	.121	.018	.212
**2-ITX-O**	**Correlation Coefficient**		1.000	.467*	.246	.275
	**Sig. (2-tailed)**		.	.021	.202	.581
**2-ITX-O_2**	**Correlation Coefficient**			1.000	.040	.131
	**Sig. (2-tailed)**			.	.627	.105
**DETX-O**	**Correlation Coefficient**				1.000	.149
	**Sig. (2-tailed)**				.	.220

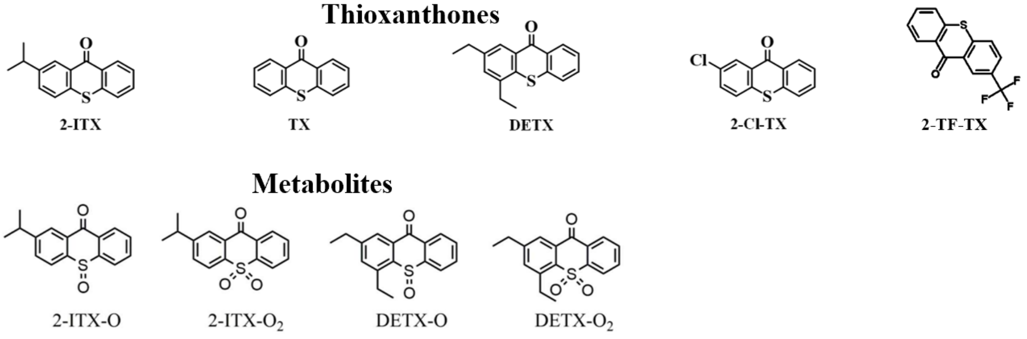

Figure S1. Chemical structure of target TXs and their metabolites analyzed in this study.

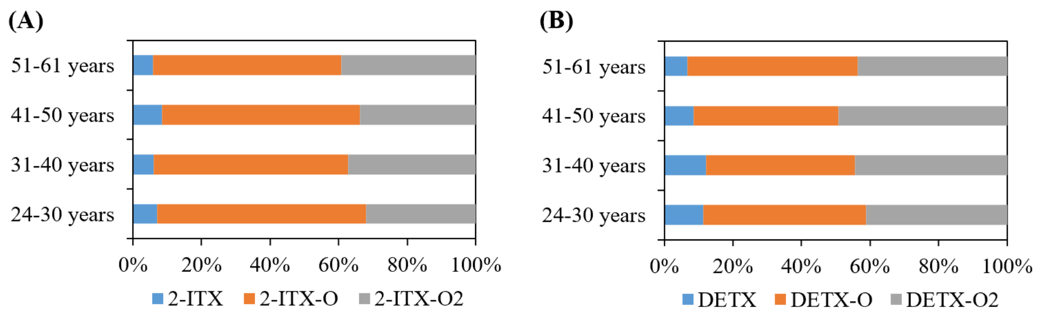

Figure S2. (A) Urinary concentration profiles of 2-ITX and its metabolites across the four human age groups. **(B)** Urinary concentration profiles of DETX and its metabolites across the four human age groups.
